# A Microfabrication Technique for High-Performance Diffractive Optical Elements Tailored for Numerical Simulation

**DOI:** 10.3390/nano15020138

**Published:** 2025-01-17

**Authors:** Xingang Dai, Yanjun Hu, Bowen Niu, Qun Dai, Yu Ao, Hongru Zhang, Gaoshan Jing, Yuan Li, Guofang Fan

**Affiliations:** 1 Key Laboratory of All Optical Network and Advanced Telecommunication Network, Ministry of Education, Institute of Lightwave Technology, Beijing Jiaotong University, Beijing 100044, China; 2 Institute of Microelectronics of the Chinese Academy of Sciences, Beijing 100029, China; 3 Shanghai Institute of Measurement and Testing Technology, National Center of Measurement and Testing for East China, National Center of Testing Technology, Shanghai 201203, China

**Keywords:** diffractive optical element, microfabrication, uniformity, photolithography

## Abstract

Diffractive optical elements (DOEs) are specialized optical components that manipulate light through diffraction for various applications, including holography, spectroscopy, augmented reality (AR) and virtual reality (VR), and light detection and ranging (LiDAR). The performance of DOEs is highly determined by fabricated materials and fabrication methods, in addition to the numerical simulation design. This paper presents a microfabrication technique optimized for DOEs, enabling precise control of critical parameters, such as refractive index (RI) and thickness. Using photolithography, we fabricated high-precision photoresist patterns on silicon and sapphire substrates, with 3 × 3 and 3 × 5 DOE beam splitter as examples. The results show a strong match between simulation and experimental data, with discrepancies of just 0.53% and 0.57% for DOE on silicon and sapphire substrates, respectively. This approach offers potential for advancing high-performance DOE devices in semiconductor manufacturing, supporting next-generation optical systems.

## 1. Introduction

Diffractive optical elements (DOE) have garnered significant attention due to their outstanding properties, such as design flexibility, compact size, lightweight nature, high diffraction efficiency, and suit for mass production [1,2,3]. DOEs are advanced optical components that precisely manipulate light diffraction through phase modulation, enabling various optical functions, including beam focusing, beam shaping, wavefront correction, and polarization manipulation [4,5]. DOE have revolutionized the fields of optics, enabled new functionalities and improved the performance of diverse optical systems, including holography, spectroscopy, augmented reality (AR) and virtual reality (VR), and light detection and ranging (LiDAR) [6,7,8,9]. However, efficiently and reliably navigating the intricate iterative process to develop high-performance DOE devices remains a challenge. This process involves crucial steps, such as optical performance evaluation, material selection, periodic diffractive structure design, fabrication, and characterization.

The optical performance of DOEs is inherently dispersive and highly dependent on the incident light wavelength, which is influenced by factors such as refractive index (RI), optical absorption, and thickness of the structured periodic elements within the material [10]. The process of realizing a high-performance DOE begins with a comprehensive understanding of the specific application requirements and performance specifications, such as applied wavelength, desired diffraction profile, and optical performance specifications. Material selection then plays a pivotal role in determining the optical diffractive efficiency, durability, and suitability of the DOE for various applications. Various materials, including BiTiO_3_ [11], polymers [12], glassy carbon [13,14], and hydrogels [15], have been utilized in DOE fabrication. Subsequently, a meticulous process of simulation and numerical analysis is employed to generate periodic diffractive structures capable of achieving the desired functionalities. This involves the application of sophisticated algorithms and methods aimed at generating optimized diffractive patterns. Critical design parameters include the spatial distribution of phase across the DOE structure surface, which dictates the behavior of diffracted light, and the selection of diffractive patterns or algorithms encoding the desired optical function [16]. Other factors, such as polarization sensitivity, angular tolerance, and fabrication constraints, are also carefully considered in the design phase to ensure the practicality devices. These algorithms and methods utilize principles of diffractive optics, numerical optimization, and computational electromagnetics, incorporating techniques like the Gerchberg–Saxton Algorithm, Iterative Fourier Transform Algorithm (IFTA), Binary Phase-Only Filter (BPOF), and Convolutional Neural Networks (CNNs) [17,18,19,20,21]. Once the DOE design phase is finalized, the focus shifts to precision fabrication at the nanoscale level, utilizing techniques such as lithography, etching [22,23,24], laser writing, holographic recording, injection molding [25], hot embossing [26], glass molding [27], and 3D printing with liquid immersion [28]. Fabrication processes must cover material properties and desired diffractive structures to ensure the creation of an accurate phase profile. Subsequent characterization of the DOE’s optical functions involves evaluating key parameters, such as diffraction efficiency and spatial resolution. For instance, conventional methods like electron beam lithography combined with plasma etching have been developed to fabricate DOEs with enhanced resolution. Dong Cheon Kim et al. fabricated a 7 × 7 beam splitter by reactive ion etching after photolithography with a calculated and measured uniformity difference of 7.00% and a total diffraction efficiency difference of 1.66%, respectively [29]. To reduce DOE structure surface roughness, Huang Hao et al. innovatively employed lithography alongside a twice-imprinting technique to design and fabricate a wide-angle 5 × 5 beam splitter without utilizing etching for creating DOE surface morphology, with a final design and test uniformity difference of 8.80% and a total diffraction efficiency difference of 3.00%, respectively [30]. Reut Kedem Orange et al. introduced a 3D-printing approach for DOEs, which involves the integration of two nearly RI-matched transparent materials [28]. This method offers considerable scalability in the axial dimension while enhancing control over thickness during the manufacturing process. Variations during fabrication techniques, especially fidelity and surface roughness of intricate micro and nanostructures, significantly influence DOE performance. Achieving a close match between simulated and fabricated DOE optical functions continues to be a challenging and iterative process for developing high-performance DOE.

This paper introduces a microfabrication technique for fabricating DOE tailored for numerical simulation. Using a DOE beam splitter as an example, we demonstrate the design and fabrication process. The DOE beam splitter array is designed with photoresist patterns on silicon and sapphire substrates, using the IFTA. A precision photolithography process is employed to control the refractive index (RI) and thickness of the DOE, in alignment with simulation results. Experimental measurements show excellent agreement between simulation and fabrication, with maximum deviations of 0.53% and 0.57% for silicon and sapphire substrates, respectively. This approach offers significant potential for advancing DOE devices, enabling the development of next-generation optical systems with high efficiency and reliability.

## 2. Materials and Methods

**Figure 1:**
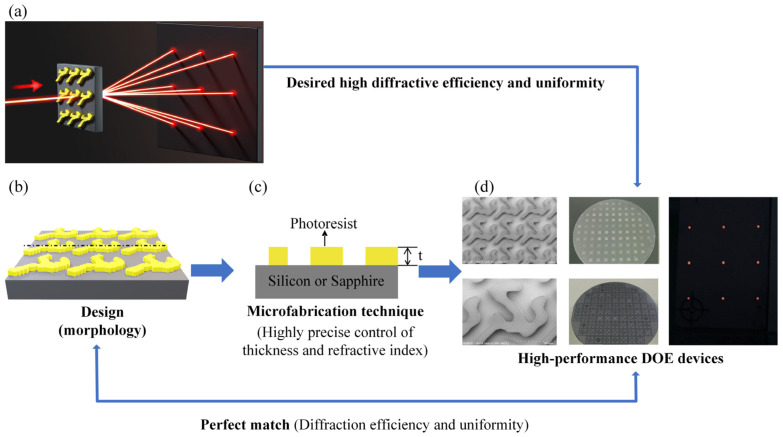
(**a**) Schematic diagram of DOE beam splitter. (**b**) Design of DOE morphology. (**c**) Well-controlled microfabrication technique with precise control of the thickness and RI. (**d**) Characterization of high-performance DOE devices, precisely tailored through numerical simulation.

The entire iterative process is illustrated in Figure 1. The whole iterative process includes several crucial steps, such as periodic diffractive structure design, microfabrication to enable precise control of the RI and the thickness of DOE, and characterization.

### 2.1. Optical Performance Evaluation

In the process, a DOE beam splitter is taken as an example, which is designed to split a single laser beam into a uniform beam spot array, with each spot retaining the characteristics of the origin beam. A DOE beam splitter can generate either a 1-dimensional beam array (1 × *N*) or a 2-dimensional beam matrix (*M* × *N*), depending on the designed diffractive pattern of the DOE device.

Total diffraction efficiency (*η*) and uniformity (*σ*) are the two important parameters to determine the performance of DOE beam splitters. The total efficiency (*η*) and uniformity (*σ*) of DOE are quantified by Equations (1) and (2) as follows:

(1) $$\eta =\sum_{i=0}^{m-1}\eta_{i}=\sum_{i=0}^{m-1}\frac{P_{i}}{P},$$

(2) $$\sigma =\frac{P_{max}-P_{min}}{P_{max}+P_{min}},$$

where *P_i_* and *P* represent the energy of each order emerging from beam splitter and the energy of the light source, respectively; *η_i_* is the *i*th order diffraction efficiency; *m* denotes the total order of the beam splitter; and the *P_max_* and *P_min_* represent the maximum and minimum power of the beam splitter, respectively.

### 2.2. Material Selection of DOE

A two-layer DOE beam splitter, composed of DOE morphology layer and substrate layer is proposed. The substrate layer supports DOE morphology, and the DOE morphology layer performs the beam-splitting function.

For substrate material, the silicon and sapphire wafer are chosen as a comparison. The silicon is a material commonly used in semiconductor processing. However, silicon as the substrate material has lower transmittance in the infrared band. The sapphire can offer higher transmittance in the infrared band.

For the DOE morphology layer, we chose AZ 12XT-20PL-10 photoresist (MERCK KGaA, Darmstadt, Germany) due to versatility in achieving thickness ranging from 4 μm to 14 μm by simply adjusting the spin-coating speed.

### 2.3. Periodic Diffractive Structure Design

The DOE structures were designed with an IFTA [19,31]. This algorithm iteratively adjusted the phase profile of DOE to match the desired intensity distribution in the output plane. The IFTA procedure for DOE design is shown as the followed:

(1) Initialization: The amplitude and wavelength of incident light are inputted, and a random initial phase profile is generated. At this stage, the incident light is the key factor, while the initial phase profile is arbitrary. The generated phase profile is a two-dimensional array of phase values.

(2) Forward propagation: The distribution of observed plane field is obtained by calculating the Kirchhoff diffraction integral using Fourier transform.

(3) Error calculation: The error function is calculated by comparing the intensity distribution obtained from the Fourier transform with the desired intensity distribution. This function quantifies the discrepancy between the actual and desired optical fields.

(4) Amplitude replacement: The amplitude of the field obtained in step 2 is replaced by the desired intensity distribution (i.e., a uniform distribution of the spot array). This new field function is then combined with the phase profile from step two.

(5) Backward propagation: The distribution of the source plane field is obtained by calculating the inverse Kirchhoff diffraction integral using a Fourier transform of the function obtained in step four.

(6) Iteration: The phase profile from step 5 is kept unchanged, while the field amplitude is replaced with the amplitude of the incident light. Steps 2 through 6 are repeated until the change in the error function is minimal, at which point the iteration stops, and the desired phase profile is obtained.

(7) Finalization: Once convergence is achieved, the design of the DOE is finalized by extracting the optimized phase profile. This optimized phase profile represents the diffractive pattern that should be fabricated onto the DOE substrate to achieve the desired optical functionality.

In this paper, the chosen parameters include a wavelength of 1550 nm and a resolution of 1 μm. A random initial phase distribution was used to avoid local minima.

### 2.4. Microfabrication Technique

**Figure 2:**
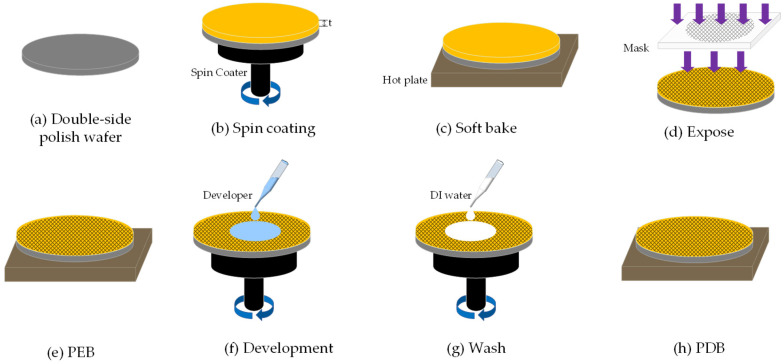
Microfabrication process of DOE.

The microfabrication process for the DOE is illustrated in Figure 2. Step (a): beginning with double-side polish, the wafer is cleaned and double-side polished with an average roughness of less than 0.5 nm. Step (b): the material of DOE morphology layer is spin-coated onto the double-side polished wafers, starting at 500 rpm for 5 s with a ramp rate of 100 rpm/s, followed by an increase to 3200 rpm for 30 s at a ramp rate of 9000 rpm/s (Cee^®^ 200 CBX, Brewer Science, INC., Rolla, MO, USA). Step (c): the film was soft-baked at 110 °C for 2 min on a vacuum hot plate. Step (d): the DOE film is exposed by the broadband UV with a dose of 113 mJ/cm^2^ using a contact aligner (ABM/6/500/NUV/DCCD/M, ABM, San Jose, CA, USA). Step (e): post-exposure baking (PEB) is carried out at 90 °C for 90 s. Step (f): development is then conducted using AZ 300 MIF (MERCK KGaA, Darmstadt, Germany), which dissolves the UV-exposed areas. During development, the process began with spin-coating AZ 300 MIF at a low speed of 40 rpm for 5 s, with a ramp rate of 900 rpm/s. After setting, development proceeded for 22 s. The film was subsequently spun at 500 rpm for 5 s with an acceleration of 900 rpm/s. This sequence was repeated for two development cycles. Step (g): the remaining developer solution is washed away using water, and the excess water is removed using nitrogen. Step (h): the developed wafer is subjected to post development baking (PDB).

### 2.5. Charzcterization of DOE

**Figure 3:**
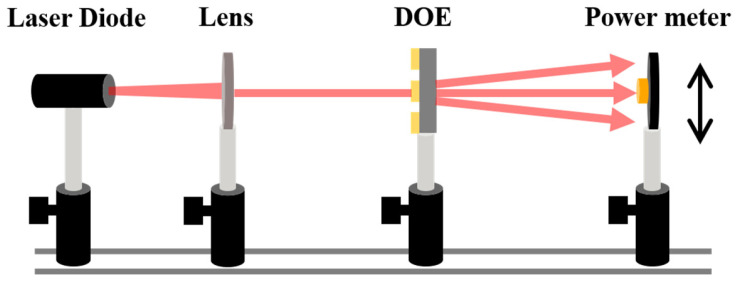
Schematic of measurement set-up for DOE beam splitter.

To characterize the fabricated DOE beam splitter, a set-up system was configured to measure the diffractive efficiency of beam array. As illustrated in Figure 3, the setup includes a laser diode (ML925B45F, Thorlabs, Newton, NJ, USA) with a center wavelength of 1550 nm, collimated aspheric lens (C660TME-C, Thorlabs, Newton, NJ, USA) within the wavelength range of 1050–1700 nm, and a power meter (PM122D, Thorlabs, Newton, NJ, USA) with a photodiode power sensor (S122C, Thorlabs, Newton, NJ, USA).

## 3. Results and Discussion

A DOE beam splitter is highly dispersive, and the feature size of the DOE microstructure is closely related to the incident wavelength. To simplify the fabrication technique and measurement system, we selected an incident laser wavelength of 1550 nm. This wavelength is close to the critical dimension of one micron in contact lithography, and the corresponding laser source, optical elements, and photodetector are commonly used in the optical communication field. The 3 × 5 and 3 × 3 DOE beam splitters are taken as an example to investigate the microfabrication technique tailored for numerical simulation of DOE.

### 3.1. Design of the DOE Beam Splitter

In this design of 3 × 3 and 3 × 5 DOE beam splitters using IFTA, the pixel resolution is 1 μm × 1 μm, the grating period is 90 μm × 90 μm, and the phase matrix size is 90 μm × 90 μm. The beam splitter spots are 700 mm from the DOE with 12 mm between each order spot.

**Figure 4:**
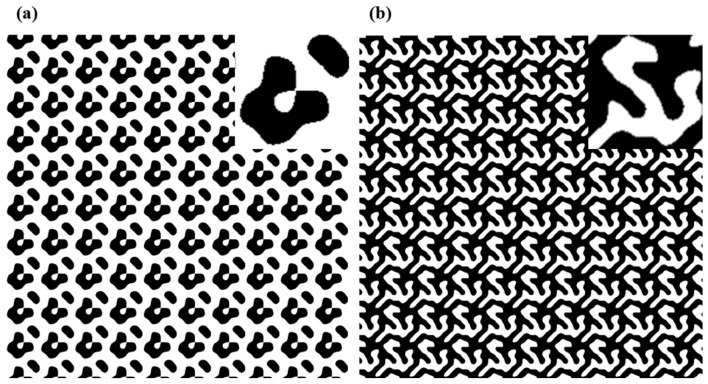
The binary phase distribution profile (phase π in white, phase 0 in black) of DOE designed using IFTA: (**a**) the phase distribution profile of 3 × 5 DOE beam splitter; (**b**) the phase distribution profile of 3 × 3 DOE beam splitter. Top insets: magnified image of a unit cell.

The binary phase (0–π) distributions of DOE beam splitters designed by IFTA is shown in Figure 4 for 3 × 3 and 3 × 5 DOE splitters. The simulation results show that the total efficiency and uniformity of the DOE beam splitter, without considering the wafer (when light is transmitted through the wafer, the energy is reduced thus lowering the total diffraction efficiency), are 65.56% and 12.58% for the 3 × 3 DOE and 74.85% and 4.32% for 3 × 5 DOE under the ideal condition. The ideal condition requires the relation of the thickness and RI of the DOE morphology layer to meet the following equation [32]:

(3) $$t=\frac{k\lambda }{2(n-1)},$$

where *t* represents the thickness of the DOE morphology layer; *k* is a positive odd integer; *λ* denotes the wavelength of the incident light; and *n* denotes the RI of the DOE morphology layer.

This equation highlights that the RI and thickness of morphology layers are critical parameters for the DOE beam splitter. In other words, the optical performance of DOEs is inherently dispersive and highly dependent on the incident light wavelength, RI, thickness of the morphology layer, and optical absorption of the substrate material. The proposed microfabrication technique is developed to tailor for numerical simulation by meeting the Equation (3) for the optimal performance of DOE.

### 3.2. Refractive Index of the Morphology Layer

**Figure 5:**
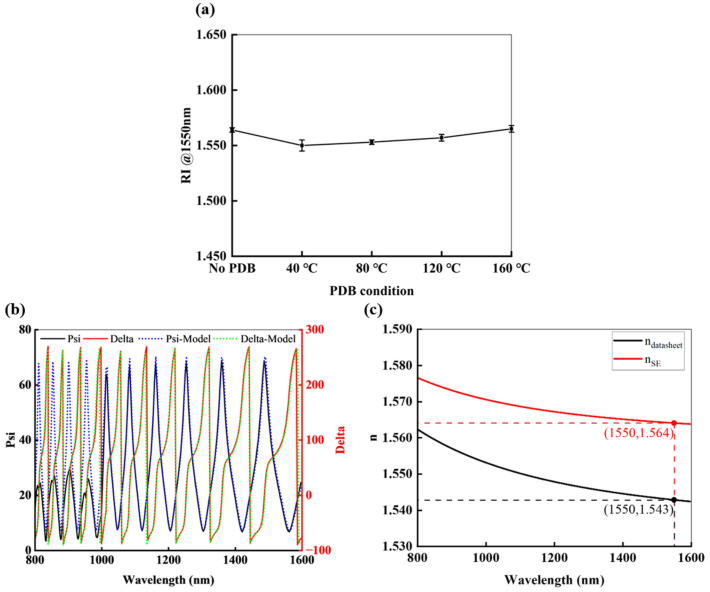
RI of photoresist as the morphology layer. (**a**) The RI of the photoresist under different PDB conditions; (**b**) Psi and Delta results obtained by SE and fitted using the Cauchy model without PDB; (**c**) Comparison of the RI from the SE measurement and the RI provided by datasheet.

The RI of the morphology layer is crucial to the performance of the DOE beam splitter, making it the first key parameter to determine. Spectroscopic ellipsometry (SE) is a non-destructive optical technique that analyzes the change in polarization of light upon reflection to determine a material’s optical properties and thickness [33]. Psi and Delta are the crucial parameters of the SE. Psi represents the amplitude ratio between the p- and s-polarized light, and Delta represents the phase difference between the p- and s-polarized light. The refractive index and thickness of the measured materials can be obtained by fitting the model with the Psi and Delta. The RI of photoresist as the DOE morphology layer is investigated under a different process condition, which is measured using spectroscopic ellipsometry (V-VASE, J.A. Woollam, Lincoln, NE, USA), and compared with the RI provided by the datasheet. The results are shown in Figure 5.

Figure 5a shows the RI of the photoresist under different PDB conditions (performed five minutes at 40 °C, 80 °C, 120 °C, and 160 °C, respectively). One can observe that any change in the process leads to RI variations. This further confirmed the importance of precise RI measurement.

Figure 5b presents the Psi and Delta results obtained from SE for the photoresist of morphology layer without PDB. The results are fitted using the Cauchy model. The fit is perfect in the wavelength range of 1200 nm to 1600 nm, which means that the SE measurement is the precise method for the RI of material. The RI measured by SE should be an acritical parameter for the well-controlled microfabrication technique to realize high-performance DOE devices.

Figure 5c compares the RI of photoresist measured by the SE with the RI of photoresist provided by datasheet. There is a big difference for the refractive indices from the SE and datasheet. The RI difference of the SE and datasheet shows an error of 1.36%. According to Equation (3), this means the deviation of the ideal thickness of DOE is 0.2657 μm using RI from the SE and datasheet, which significantly affects subsequent fabrication. This illustrates the importance of precise RI measurement for the proposed microfabrication technique to realize high-performance DOE devices. One can obtain that the RI of the morphology layer is 1.564 for the wavelength of 1550 nm in our discussion.

### 3.3. Thickness of the Morphology Layer

After the RI of the morphology layer is determined to be 1.564, the fabricated ideal thickness of the morphology layer can be determined to be *t_ideal_* = 6.8706 μm according to Equation (3). The precise control and measurement of the thickness of the morphology layer will be crucial for high-performance DOE devices. The thickness of photoresist as the DOE morphology layer is investigated under different process conditions.

**Figure 6:**
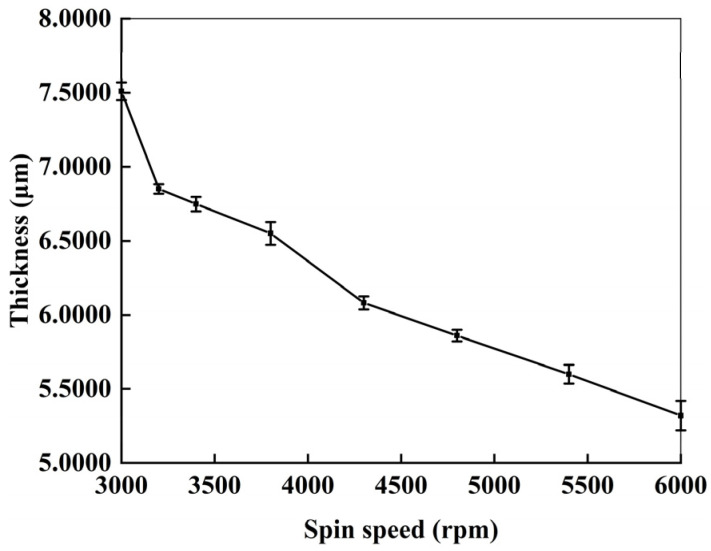
Dependence of photoresist thickness on spin-coating speeds.

The thickness with the spin-coating speed is explored as shown in Figure 6. One can find that the thickness of photoresist gradually decreases with the increase in the spin-coating speed. The ideal thickness can be obtained by well-control of the spin-coating speed. When spin-coating speed is 3200 rpm, the thickness of photoresist is near 6.8 μm, which is consistent with the ideal thickness of DOE beam splitter. Therefore, we employed a spin-coating speed of 3200 rpm for the subsequent fabrication.

**Figure 7:**
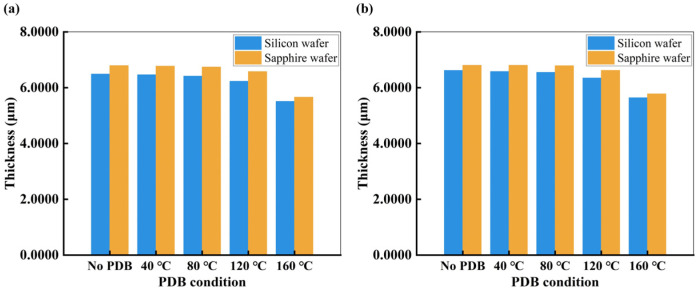
(**a**) Thickness measurements of a single DOE on the silicon and sapphire wafer under different PDB conditions; (**b**) Thickness measurements of the whole silicon and sapphire wafer under different PDB conditions.

After spin-coating, the PDB is another step followed to determine the thickness. The thickness will change with the different temperature of PDB as shown in Figure 7. The thickness of the fabricated DOE is measured using a stylus profilometer (DektakXT, Bruker, Billerica, MA, USA).

Figure 7a shows the average thickness (five measurements within a single DOE) of single DOE at different PDB temperatures. It can be seen from Figure 7a that the thickness of the photoresist on the silicon wafer is lower in the photoresist on the sapphire wafers due to silicon having a higher thermal conductivity, resulting in more volatilization of the photoresist on the silicon wafer. Figure 7b shows the average thickness (five measurements for different locations of DOE on the wafer) of whole wafer at different PDB temperatures. As can been seen in Figure 7, the thickness of photoresist decreases with increasing PDB temperature, which is because more of the solvent of the photoresist evaporates as the temperature of the PDB increases. This shows the PDB temperature is also crucial in deciding the thickness of the morphology. The temperature of the PDB is determined to ensure that the fabricated thickness is close to the ideal thickness of 6.8716 μm, and it is closest when no PDB treatment is performed.

**Figure 8:**
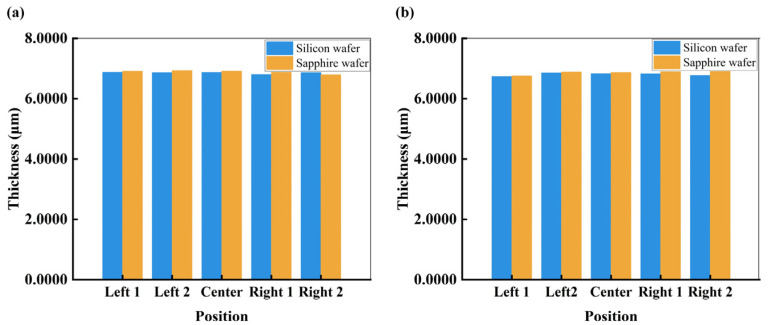
(**a**) Thickness measurement at five different positions for a single DOE on silicon and sapphire wafers. (**b**) Thickness measurements at five different positions of the whole silicon and sapphire wafers.

After PDB, the thickness of the fabricated DOE is measured using a stylus profilometer (DektakXT, Bruker, Billerica, MA, USA). Five positions (the position of center and four points uniformly from the center) are chosen to investigate the thickness of a single DOE and the whole wafer as shown in Figure 8.

Figure 8a shows the thickness of five positions (the position of center and four points uniformly from the center) on a single DOE on the silicon wafer and sapphire wafer. The coefficient variation (CV) of thickness of a DOE on silicon and sapphire wafer is 0.44% and 0.78%, respectively, indicating a high degree of stability within a DOE. The average of the five measured thicknesses is 6.8611 μm and 6.8828 μm for the DOE beam splitter on silicon and sapphire wafer, which is close to the ideal thickness of 6.8706 μm.

Figure 8b shows the thickness of five positions (the position of center and four points uniformly from the center) on the silicon and sapphire wafers. The CV of the thickness over the whole wafer is 0.68% and 0.86% for silicon and sapphire wafer, respectively, which indicates that our microfabrication technique can guarantee a small thickness error even on the whole wafer.

### 3.4. Wafer Transmittance (Silicon vs. Sapphire)

**Figure 9:**
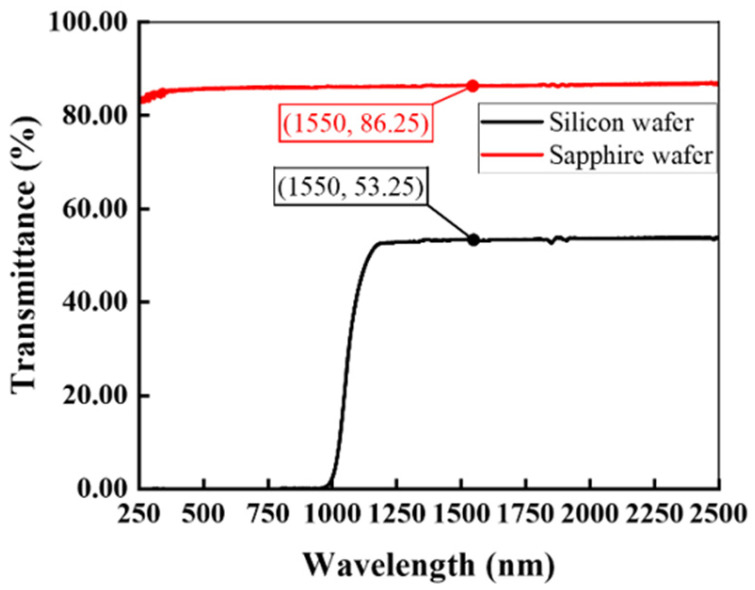
Transmittance of silicon and sapphire.

The wafer transmittance leads to direct influence on the diffraction efficiency of DOE, which should be considered to evaluate the performance of DOE. The transmittance of silicon and sapphire wafers was measured across 250–2500 nm using an Agilent Cary 5000 UV-VIS-NIR Spectrophotometer (Agilent, Santa Clara, CA, USA) as shown in Figure 9. The transmittance is 53.25% for silicon wafers and 86.25% for sapphire wafers at 1550 nm.

### 3.5. Comparison of Simulation and Experiement

**Figure 10:**
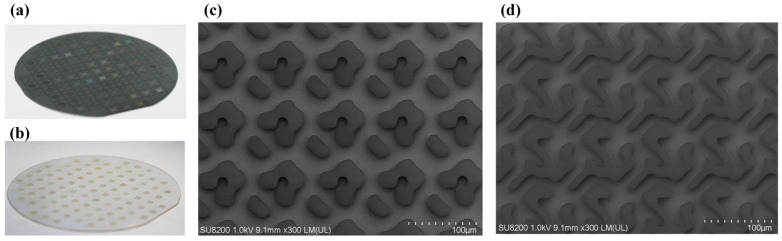
Microfabricated DOE beam splitter. (**a**) Silicon wafer. (**b**) Sapphire wafer. (**c**,**d**) Scanning electron microscopy image of the 3 × 5 and 3 × 3 DOE beam splitter.

After the periodic diffractive structure design, the microfabrication to enable precise control of the RI and thickness of DOE, an ideal DOE beam splitter is fabricated with the determined RI of 1.564, thickness of 6.8611 μm and 6.8828 μm for the DOE on silicon and sapphire wafer as shown in Figure 10. Figure 10a,b shows the DOE beam splitter on silicon and sapphire wafer, respectively. A scanning electron microscopy image of the DOE beam splitter is presented in Figure 10c.

**Figure 11:**
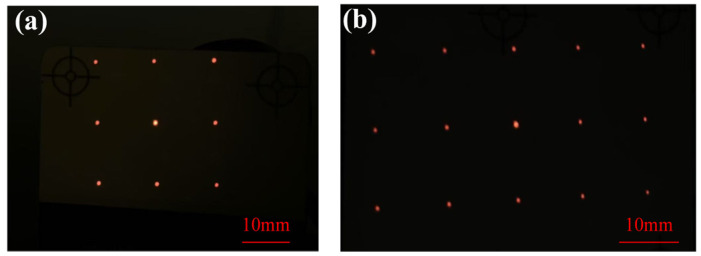
Pattern of DOE beam splitter, (**a**) 3 × 3 and (**b**) 3 × 5.

**Figure 12:**
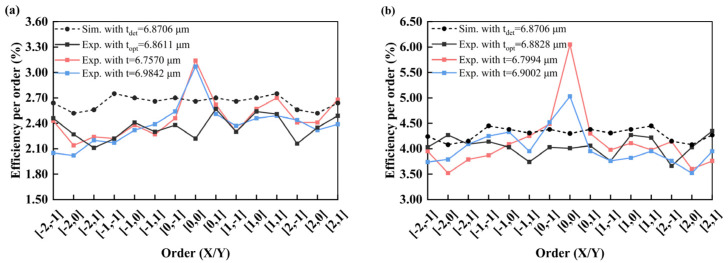
The diffraction efficiency per order of 3 × 5 DOE beam splitter, from the experimental results with different fabricated thickness and simulations results using ideal thickness, respectively. (**a**) Silicon wafer; (**b**) Sapphire wafer.

The fabricated 3 × 3 and 3 × 5 DOE beam splitters on silicon and sapphire wafer are measured using the experimental setup in Figure 3, and the results are shown in Figure 11. The measured diffraction efficiency per order of the fabricated 3 × 5 DOE beam splitter on silicon and sapphire wafer is shown in Figure 12. As a comparison, the simulation efficiency per order considered the transmittance of the wafer. Comparing the simulated and measured efficiency at per order, the measured results are slightly smaller than the simulated results. This is attributed to the fact that the theoretical simulation of light does not consider the loss due to photoresist absorption as well as the scattering from the surface of the DOE.

One can see that the experimental results with the determined thickness of 6.8611 μm and 6.8828 μm for the 3 × 5 DOE on silicon and sapphire wafer show better agreement with the simulation, compared with the experimental results with the other thickness. The efficiency per order of the DOE beam splitter with the determined thickness shows a great match between simulation and experimental results with a maximum difference of 0.53% and 0.57% for the DOE on the silicon and sapphire substrates. This highlights the importance of the control of thickness of the morphology layer for the DOE.

As a further demonstration, the total efficiency and uniformity of the DOE beam splitter with different fabricated thickness are summarized for the 3 × 3 and 3 × 5 DOE beam splitter on silicon and sapphire wafer in Table 1 and Table 2. The diffraction efficiency of the beam splitter is calculated considering the loss of light as it transmitted across the substrate. One can observe that the total efficiency and uniformity of the DOE with the determined thickness is closer to the simulation results, and better performance, compared with the DOE with the other thicknesses on silicon and sapphire wafers. This means that the proposed highly precise microfabrication technique for DOE tailored for numerical simulation works well. In addition, the measured total efficiency for all the DOEs of the determined thicknesses is about 4% smaller than that of the simulation, which can be attributed to the photoresist absorption, as well as the scattering on the DOE surface.

In a word, our proposed microfabrication method effectively controls the RI and thickness of the DOE beam splitter, achieving a perfect match between simulation and experimental results. The proposed highly precise microfabrication technique for DOEs can tailor for numerical simulation, offering valuable insights for further work and analysis.

## 4. Conclusions

In this paper, we propose a precise microfabrication method for achieving high-performance DOE tailored for numerical simulation. By carefully controlling the RI and thickness of the morphology layer, we enable accurate realization of DOE designs. The method effectively integrates crucial steps, such as optical performance evaluation, material selection, periodic diffractive structure design, fabrication, and characterization.

Using the DOE beam splitter on the silicon and sapphire wafer as examples, we demonstrate the application of this microfabrication technique. The photolithography process ensures precise control of both RI and thickness, closely aligning with the simulated results. Experimental measurements show excellent agreement with simulations, with deviations of only 0.53% and 0.57% for silicon and sapphire substrates, respectively. This method holds significant promise for advancing DOE devices, paving the way for the development of next-generation optical systems and applications with high efficiency and reliability.

## Figures and Tables

**Table 1:** Total efficiency (η) and uniformity (σ) of 3 × 3 and 5 × 5 DOE beam splitter on silicon wafer.

DOE Types	Simulation and Experiment of DOE	Thickness (μm)	η (%)	σ (%)
3 × 3	Simulation	6.8706 (ideal)	34.91	12.58
	Experiment	6.8272 (determined)	30.64	18.84
		6.8044	30.89	24.72
		6.9805	31.71	29.88
3 × 5	Simulation	6.8706 (ideal)	39.72	4.34
	Experiment	6.8611 (determined)	35.29	9.83
		6.7570	36.97	18.94
		6.9842	35.74	20.63

**Table 2:** Total efficiency (η) and uniformity (σ) of 3 × 3 and 5 × 5 DOE beam splitter on sapphire wafer.

DOE Types	Simulation and Experiment of DOE	Thickness (μm)	η (%)	σ (%)
3 × 3	Simulation	6.8706 (ideal)	56.55	12.58
	Experiment	6.8947 (determined)	52.61	15.42
		6.8401	52.69	16.73
		6.9001	52.76	20.61
3 × 5	Simulation	6.8706 (ideal)	64.31	4.34
	Experiment	6.8828 (determined)	60.69	8.61
		6.9002	60.41	17.66
		6.7994	61.88	26.44

## Data Availability

Data will be made available upon request.
